# Soft electroceutical immunomodulation

**DOI:** 10.1093/nsr/nwag009

**Published:** 2026-01-08

**Authors:** Yuze Zheng, Guangqing Yang, Bozhi Tian

**Affiliations:** Department of Chemistry, The University of Chicago, USA; Department of Chemistry, The University of Chicago, USA; Department of Chemistry, The University of Chicago, USA

Bioelectronic medicine [1–3] has long promised a future in which diseases are treated not by systemic drugs, but by precisely modulating the body’s own electrical language. At the heart of this vision lies a fundamental challenge: biology is soft, dynamic and wet, while traditional electronics are rigid, static and dry. Over the past decade, hydrogel-based bioelectronics [4,5] has begun to close this gap. Conductive hydrogels—rich in water, mechanically compliant and capable of both ionic and electronic transport—have emerged as a new class of materials that can speak the native language of tissues. Early successes demonstrated injectable electrodes, adhesive interfaces and soft neural contacts, yet chronic, organ-specific neuromodulation has remained elusive, especially for fragile, irregular nerves embedded deep within moving organs.

**Figure 1.**
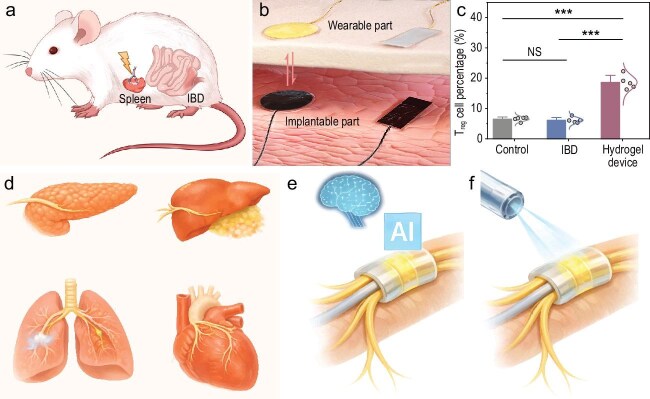
Soft electroceutical immunomodulation. (a) Conceptual schematic of wireless splenic nerve stimulation enabling electroceutical treatment of inflammatory disease by restoring immune balance. Soft, fiber-based electrodes conformally interface with peripheral neurovascular structures using adhesive fixation. (b) Illustration of a fully implantable, wireless neuromodulation system in which subcutaneous electrodes couple with external wearable components to enable power delivery without batteries or hard wiring. (c) Representative immune outcome showing restoration of regulatory immune cell (T_reg_) populations following neuromodulation treatment. (d) Extension of this modular platform to other peripheral neural targets for regulating metabolic, inflammatory, pulmonary and cardiac functions. (e) Integration with biosensing and intelligent control to enable adaptive, closed-loop electroceutical therapies. (f) Coupling soft biointerfaces with optical or photoelectrochemical actuation to achieve spatially precise, minimally invasive modulation. (a–c) are adapted from Liu *et al*. [6].

Luo and his team [6] address this challenge by rethinking bioelectronics from the material up to the therapeutic outcome. Rather than adapting rigid devices to soft biology, they construct a fully hydrogel-based, wireless neurostimulator that behaves more like living tissue than a machine (Fig. 1). At the core of their approach is a carefully engineered conductive hydrogel produced through a glutaraldehyde–salt–wet annealing strategy. This process resolves a long-standing trade-off in the field: achieving ultrahigh electrical conductivity without sacrificing mechanical softness. The resulting hydrogel combines tissue-matched compliance with conductivities rivaling metallic systems, enabling stable, long-term electrical coupling *in vivo*.

The story deepens at the interface. Instead of planar or cuff-style electrodes that compress or damage nerves, Luo and his team introduce fiber-shaped, bioadhesive hydrogel electrodes that gently wrap around splenic neurovascular bundles [6] (Fig. 1a). These fibers conform, adhere and move with the tissue, eliminating the need for sutures while avoiding fibrosis or immune

activation. Through hydrogel-based capacitive coupling, the device operates wirelessly and without batteries, harvesting energy transcutaneously while remaining entirely soft and implantable (Fig. 1b).

In a chronic inflammatory bowel disease [7] model, long-term splenic nerve stimulation delivered by this hydrogel system restores immune balance. Pro-inflammatory Th1 and Th17 responses are suppressed, while anti-inflammatory Th2 and regulatory T cell pathways are enhanced (Fig. 1c). Rather than blunt immune suppression, the therapy rewires neuroimmune communication, demonstrating how precision neuromodulation can reshape systemic immunity. This work moves electroceuticals beyond symptom control and toward immune reprogramming.

Looking ahead, the platform introduced by Luo and his team feels less like a single device and more like a blueprint for the future of bioelectronics. Its modular, fully soft design could be extended to other peripheral nerves (Fig. 1d) governing metabolism, inflammation and autonomic function, including targeted stimulation of the pancreatic nerve to regulate insulin secretion and glucose homeostasis, hepatic vagal branches to modulate lipid metabolism and systemic inflammation, renal nerves to influence blood pressure and fluid balance, and pulmonary autonomic fibers to control airway inflammation and bronchoconstriction. Beyond metabolic and inflammatory regulation, similar hydrogel-based interfaces could be adapted to sacral, pelvic or tibial nerves for bladder control, gastrointestinal motility and pain modulation, or to cardiac autonomic fibers for heart-rate variability and arrhythmia management. By conforming to small, irregular and moving neural structures, such platforms enable selective, organ-specific neuromodulation that is difficult to achieve with conventional cuff or rigid electrodes. Integration with real-time biosensing and AI-driven control could enable closed-loop electroceutical systems that learn, adapt and personalize therapy over time (Fig. 1e). Coupling such hydrogel interfaces with photostimulation or photoelectrochemical transduction could further enable spatially precise, minimally invasive modulation without direct electrical wiring [2,3] (Fig. 1f). Luo and his team offer a compelling glimpse of that future: one where soft, intelligent materials seamlessly integrate with living systems to restore balance, treat disease and redefine how medicine is delivered.
